# Molecular Properties of Starch–Water Interactions in the Presence of Bioactive Compounds from Barley and Buckwheat—LF NMR Preliminary Study

**DOI:** 10.3390/polym17192606

**Published:** 2025-09-26

**Authors:** Greta Adamczyk, Łukasz Masewicz, Krzysztof Przybył, Aleksandra Zaryczniak, Przemysław Łukasz Kowalczewski, Monika Beszterda-Buszczak, Wojciech Cichocki, Hanna Maria Baranowska

**Affiliations:** 1 Department of Food Technology and Human Nutrition, Institute of Food Technology and Nutrition, University of Rzeszow, 35-601 Rzeszów, Poland; 2 Department of Physics and Biophysics, Poznań University of Life Sciences, 60-637 Poznań, Poland; 3 Department of Biosystems Engineering, Poznań University of Life Sciences, 60-625 Poznan, Poland; 4 Collegium Medicum, Andrzej Frycz Modrzewski Krakow University, 30-705 Kraków, Poland; 5 Department of Food Biochemistry and Analysis, Poznań University of Life Sciences, 60-623 Poznań, Poland; 6 InnPlantFood Research Group, Poznań University of Life Sciences, 60-624 Poznań, Poland

**Keywords:** starch retrogradation, potato starch gels, low-field NMR, buckwheat hull fiber, green barley

## Abstract

The retrogradation of starch strongly influences the texture and stability of starchy foods. This study applied low-field nuclear magnetic resonance (LF NMR) to examine the effect of buckwheat hull (BH) fiber and green barley (GB) on water dynamics in normal (NPS) and waxy (WPS) potato starch gels. Relaxation times (T_1_, T_2_) and mean correlation times (τ_c_) were monitored during 15 days of storage to evaluate changes in water mobility and starch–polymer interactions. Results showed that WPS, with its high amylopectin content, retrograded earlier than NPS. The addition of BH inhibited conformational changes associated with water binding in WPS gels, indicating that insoluble fiber entrapped water within the amylopectin network. Conversely, GB promoted higher τ_c_ values in WPS, reflecting enhanced ordering and reduced water mobility, while its impact on NPS was minor. In NPS systems, BH decreased τ_c_, suggesting disruption of amylose-driven structural reorganization. These findings demonstrate that BH and GB exert opposite effects on starch retrogradation and highlight their potential as functional additives for tailoring texture and stability in starch-based food systems.

## 1. Introduction

Starch is a plant polysaccharide that functions as a reserve material, found in cereal grains, potato tubers, and legume seeds. It is one of the most abundant and versatile biopolymers in food technology, with global production exceeding 50 million tons annually [1]. As a complex carbohydrate composed of amylose and amylopectin, starch serves multiple functional roles in food systems, from basic thickening to advanced encapsulation applications [2,3]. Starch acts as a primary thickening agent across diverse food applications [2,3,4], and as a colloidal stabilizer in various food matrices [3,5]. Starch–hydrocolloid blends provide enhanced stabilization properties, with hydrocolloids increasing viscosity and inhibiting α-amylase accessibility to starch granules [5]. Starch-based particles show particular promise as stabilizers for Pickering emulsions, offering superior coalescence resistance compared to conventional emulsions [6]. Beyond basic thickening, starch significantly impacts food texture through its gelling, binding, and water retention properties [3,7]. Beyond the food sector, starch finds extensive application across diverse industrial fields [8,9,10,11].

A gel is a solid in which a spatial network formed by macromolecules extends throughout its entire volume and can be distinguished. According to the definition given by Hermans and Weidinger [12], a gel is a solid that is a homogeneous two-component system formed by a solid substance dispersed or diffused in a liquid phase. Under mechanical stress, this system behaves like a solid. The dispersed components and the solvent intermingle. This definition combines macroscopic and microscopic criteria [5]. Tests are commonly used to determine macroscopic parameters related to the mechanical deformation of the system, including the complex modulus of rigidity or viscosity [6,7]. The latter parameter in particular is difficult to define. It should be noted that such tests characterize the properties of the gel depending on the formed network. However, it does not allow for the analysis of the properties of the solvent and the polymer network at the molecular level.

An important factor influencing storage quality is starch retrogradation, which involves conformational changes in this biopolymer [2,3,4]. Starch retrogradation is a process that occurs after gelatinization, during which dispersed amylose and amylopectin molecules in starch reassociate and reorganize to form ordered structures. This process leads to notable changes in the gel’s structure and its ability to retain water [8]. During gelatinization, starch granules take in water and swell, causing amylose and amylopectin chains to disperse [9]. When cooled and stored, these chains reassemble and form new hydrogen bonds molecules. Research has shown that both the ratio of amylose to amylopectin and storage conditions (temperature, time) significantly influence the degree and rate of retrogradation. Structural rearrangement during retrogradation affects the nutritional properties, texture, and shelf life of starchy foods [10,11,12]. Controlling starch properties to modify the retrogradation process is essential in food science for optimizing product quality [13,14,15].

In research on starch retrogradation, both high-resolution and low-field NMR methods provide information on the reorganization of amylose and amylopectin chains, water interactions with starch molecules, and how these changes affect gel structure and water-binding capacity [16,17,18,19,20,21,22]. The low-field NMR method involves measuring the spin–lattice and spin–spin relaxation times of protons present in the system. In biological systems such as biopolymer gels, dipole interactions play a significant role in relaxation processes [23,24,25,26,27,28,29].

The effect related to the presence of water is the attachment or detachment of protons by functional groups due to hydration. Rotationally bound water molecules can form characteristic hydration shells. Water bonding is much weaker than that of structural water molecules. As a result, it is easier to break such bonds and create new ones with different water molecules [30,31]. Therefore, there is a continuous exchange of water molecules between the hydration layer and unbound water. This process, called chemical exchange, is the main factor in averaging the dynamic parameters related to the molecular movement of water molecules. This can be seen in relaxation studies using nuclear magnetic resonance. Water molecules can also be found near the hydrophobic areas of microparticles, causing a local change in the relaxation time of water molecule reorientation in the liquid phase.

The speed of molecular movements in a system has a significant impact on the speed of relaxation processes [18]. The spin–lattice relaxation time T_1_ provides information about the conformational and dynamic properties of the system. The faster the movement, the higher the T_1_ relaxation time values. This is due to the weakening of dipole interactions [19]. Relaxation times are macroscopic parameters, which in turn can be linked to parameters that determine molecular movements in the system.

Buckwheat hull and green barley represent promising plant-based ingredients with considerable nutritional and functional potential for food applications. Buckwheat is a rich source of dietary fiber, phenolic compounds, and minerals, providing strong antioxidant capacity and potential health benefits linked to reduced risk of metabolic disorders [32,33,34,35,36,37,38,39]. In turn, green barley contains bioactive compounds such as vitamins, chlorophyll, flavonoids, and essential amino acids, which contribute to its antioxidant, anti-inflammatory, and detoxifying properties [40,41,42]. Beyond their nutritional value, both materials may also modulate water interactions and structural organization in starch-based systems, thereby influencing texture, stability, and retrogradation behavior. Their incorporation as natural additives align with the current demand for functional ingredients that support both health promotion and the technological improvement of food products.

Previous research by Sikora et al. [43] showed that analyzing the molecular properties of water in starch gels from different botanical origins significantly broadens our understanding of biopolymer–water interactions and offers opportunities to explain the processes occurring during retrogradation. Furthermore, the study showed that buckwheat hull fiber had a greater effect on the pasting, flow and texture properties of natural potato starch than on waxy starch and exhibited a stronger interaction with amylose than with amylopectin [44]. However, in research on starch gel with green barley, it was found that the addition of extract from young barley plants causes a decrease in the measured strength of the gels, affecting the viscous properties of these samples [45]. Additionally, the storage time of the gels obtained with barley extract influences the increase in the elastic properties of the samples [44,45].

The aim of the research was to analyze the effect of fiber additives, such as buckwheat hull fiber and lipolyzed green barley on the retrogradation processes of both normal and waxy potato starch. The selection of two types of starch—normal and waxy—was intended to verify whether and how the amylopectin content, compared to amylose, changes the interaction possibilities of bioactive compounds with biopolymer in the presence of water. Another focus was to analyze the influence of amylose on retrogradation processes in the presence of such compounds.

Gels containing 5% (*w*/*w*) mixtures of potato starch and fiber preparations were studied using the LF NMR method. This is a non-invasive and non-destructive technique that enables long-term examination of the same sample.

## 2. Materials and Methods

### 2.1. Materials and Chemicals

Normal (non-waxy) potato starch (NPS) was obtained from Przedsiębiorstwo Przemysłu Ziemniaczanego Bronisław Sp. z o.o. (Strzelno, Poland), while waxy potato starch (WPS) was obtained from AVEBE FOOD (Veendam, The Netherlands). Ecological dietary fiber separated from roasted buckwheat hulls (BH) was purchased from Look Food (Lublin, Poland) and green barley (GB) from LyoFood (Kielce, Poland).

### 2.2. Gels Preparation

The study was performed on either 5 w% starch paste samples (denoted as NPS and WPS for non-waxy starch and waxy starch pastes, respectively) or samples composed of 4.8 w% starch and 0.2 w% fibers added (NPS/BH and NPS/GB for NPS with buckwheat hull fiber an green barley; WPS/BH and WPS/GB, respectively for WPS). The slurry of starches and their blends with fibers were heated on a gentle stirring for 30 min at 90 °C. Resulting hot pastes were passed into measurement viols (0.2 cm^3^), closed with Parafilm^®^ and allowed to stand for cooling to room temperature. So thermally equilibrated samples in the measurement viols were cooled to 5 °C in an ice shrank.

### 2.3. Determination of Amylose Content

Amylose content in the starch samples was determined using the method of Morrison and Laignelet [46]. Absorbance of the iodine–starch complex was measured at 635 nm using a Spectroquant Pharo 300 spectrophotometer (Merck, Darmstadt, Germany). Each sample was analyzed in triplicate.

### 2.4. LF NMR Relaxometry

Relaxation times were measured after 0, 1, 4, 7, 9, 11 and 15 days of storage at 5 °C. The spin–lattice (T_1_) and spin–spin (T_2_) relaxation times were taken at 20 MHz using a PS20 impulse ^1^H NMR spectrometer (ELLAB, Poznań, Poland) equipped with an integrated temperature control system. Before the experiments, samples placed in the spectrophotometer were allowed to reach 20 °C.

The inversion-recovery (π – t − π/2) pulse sequence was applied for taking T_1_ relaxation times. Distances between impulses (t) were changed within the range from 100 to 1000 ms at the 20 s repetition time. Each time, 32 FID signals and 110 points from each FID signal were collected.

Calculations of the spin–lattice relaxation time values were performed using the CracSpin software [47]. That software provided calculations of relaxation parameters from experimental data using a “spin grouping” approach. Marquardt’s method of minimization was applied for fitting multiexponential decays [48]. The accuracy of the relaxation parameters was estimated, and the standard deviations were given. Time changes of the current value of the FID signal amplitude in the employed frequency of impulses are described by Equation (1):

(1) $$M_{z}\left(t\right)=M_{0}\left(1-{2e}^{\frac{-t}{T_{1}}}\right)$$

where M_z_(t) is the actual magnetization value, M_0_ is the equilibrium magnetization value, t is the distance between impulses, and T_1_ is the spin–lattice relaxation time.

Measurements of the T_2_ spin–spin relaxation times were taken using the pulse train of the Carr–Purcell–Meiboom–Gill spin echoes (π/2 – TE/2 – (π)_n_) [49,50]. The distance between π (TE) impulses was 2 ms and the repetition time was 20 s. The number of spin echoes (n) reached 100. Tree accumulation signals were employed. The spin–spin relaxation time values involved an adjustment of values of the echo amplitudes to Equation (2):

(2) $$M_{x,y}\left(TE\right)=M_{0}\sum_{i=1}^{n}p_{i}e^{\frac{-TE}{T_{2i}}}$$

where M_x,y_ (TE) is the echo amplitude; M_0_ is the equilibrium amplitude; TE is the distance between π impulses; p_i_ is the fraction of protons relaxing with the T_2i_ spin–spin relaxation time.

Calculations were carried out with TableCurve v5.1. The spin-echo decays were fitted to Equation (2) by nonlinear least-squares, and the precision of the resulting relaxation parameters was quantified by their standard deviations.

A monoexponentially magnetization recovery and spin-echoes delay was found, which meant that the system relaxed within one T_1_ spin–lattice relaxation time and one T_2_ spin–spin relaxation time.

## 3. Results and Discussion

Amylose content exerts a decisive influence on the retrogradation behavior of starch pastes by modulating both the rate and extent of molecular reassociation. During gelatinization, amylose readily leaches from granules and, due to its relatively linear structure, rapidly forms double helices and crystallites upon cooling, resulting in a firm gel network, increased initial hardness, and pronounced syneresis [51,52]. The starches used in the study contained amylose at levels of 26.72% and 9.27% for NPS and WPS, respectively.

The analysis of relaxation times, presented in Table 1, revealed that NPS starch gels exhibited longer relaxation times compared to WPS gels. This difference is directly attributable to the distinct amylose and amylopectin concentrations present in the two starch types. A reduction in relaxation time indicates the entrapment of water molecules within the polymeric network [53]. However, it cannot be unequivocally determined whether the water interacts directly with specific sorption sites on the starch matrix or remains confined within intermolecular voids of the network.

Based on the measured relaxation times T_1_ and T_2_, the average correlation times (τ_c_) were calculated. For this purpose, the Bloembergen–Purcell–Pound (BPP) equations [54,55] describing the relationship between the macroscopic relaxation times (T_1_ and T_2_) and the microscopic average correlation time (τ_c_) were analytically solved with respect to the parameter τ_c_.

(3) $$\frac{1}{T_{1}}=\frac{6}{20}\frac{\mu_{0}^{2}}{16\pi^{2}}\frac{\gamma^{4}\hbar^{2}}{r_{0}^{6}}\left[\frac{\tau_{c}}{1+{\left(\varpi \tau_{c}\right)}^{2}}+\frac{4\tau_{c}}{1+{\left(2\varpi \tau_{c}\right)}^{2}}\right]$$

(4) $$\frac{1}{T_{2}}=\frac{3}{20}\frac{\mu_{0}^{2}}{16\pi^{2}}\frac{\gamma^{4}\hbar^{2}}{r_{0}^{6}}\left[3\tau_{c}+\frac{5\tau_{c}}{1+{\left(\varpi \tau_{c}\right)}^{2}}+\frac{2\tau_{c}}{1+{\left(2\varpi \tau_{c}\right)}^{2}}\right]$$

where μ_0_ is the permittivity of free space, γ is the magnetogyric ratio, h is the Planck constant, r_0_ is the distance of the interacting nuclei, and ω is the resonance frequency (ω = 2πf, f is the Larmor frequency, the spectrometer frequency).

**Figure 1:**
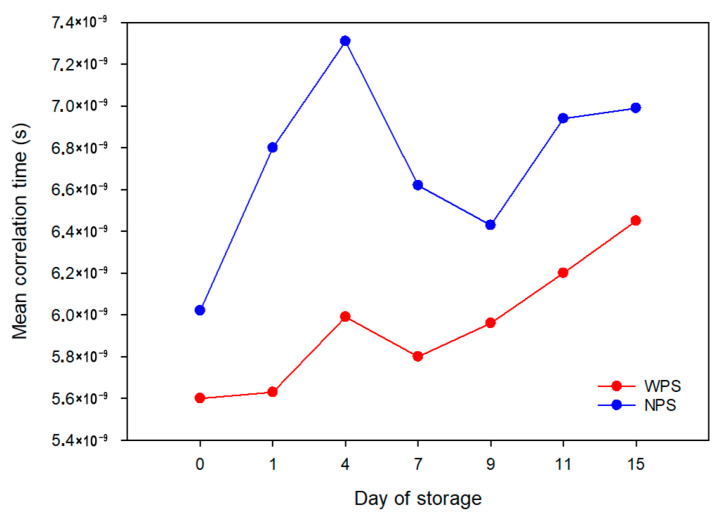
Calculated mean correlation times for NPS and WPS gels during storage.

The determination of mean correlation times enables the analysis of water molecular dynamics within the system [56]. During the 15-day observations (Figure 1), it was found that NPS forms a gel in which water molecules exhibit more restricted rotational mobility compared to the WPS gel. Thus, it can be concluded that an increased content of branched polymers (amylopectin) reduces starch–water interactions, which is manifested by a greater freedom of water molecular motions [57,58].

The results obtained during 15 days of storage of NPS and WPS gels clearly demonstrate distinct retrogradation dynamics between the two systems. In WPS, retrogradation is initiated after approximately 7 days, as evidenced by the monotonic increase in mean correlation times, which reflects progressive structuring of the gel matrix and a concomitant reduction in water mobility. In contrast, NPS, with its higher amylose content, initially exhibits strong water binding during the early storage period (up to 4 days), followed by a gradual redistribution of water molecules into interchain polymeric domains. A marked increase in mean correlation time is only observed after 11 days of storage, indicating that the immobilization of water molecules due to retrogradation is significantly delayed compared to WPS. These findings highlight the crucial role of amylose in modulating water–polymer interactions and underscore the slower structural reorganization characteristic of amylose-rich gels [59,60].

Recent research conducted by Adamczyk et al. in 2019 and 2023 indicates that native starches, due to their higher amylose content (normal potato starch PS contains approximately 29% amylose), exhibit a faster and more noticeable increase in gel hardness over time compared to waxy starches (WPS, <1% amylose) [61,62]. This is related to the retrogradation process, where short-term changes (up to 48 h) in the gel structure are caused by amylose, while amylopectin is responsible for long-term retrogradation (lasting several weeks). Waxes (WPS) show greater restriction of water molecule mobility compared to normal starch gels (NPS), which can be attributed to the higher proportion of branched amylopectin chains [61,62].

Building on the previous findings, the subsequent stage of the study focused on evaluating the effect of incorporating 0.02% BH and GB on water-binding dynamics in NPS and WPS gels. The results obtained from this investigation provide valuable insights into the potential of such functional additives to modulate gel properties and, in particular, to mitigate retrogradation processes, thereby expanding their prospective applications in starch-based systems.

The addition of both preparations was found to increase T_1_ values in NPS and WPS gels, although the most pronounced changes during storage occurred in gels containing GB. This finding may indicate that water molecules are less efficiently entrapped within the starch network when the additives are present, leading to shorter spin–lattice relaxation times (T_1_) in systems with reduced starch content. Distinct differences in spin–spin relaxation times (T_2_) were also observed between systems with BH and GB. In WPS gels, incorporation of either preparation (BH or GB) extended relaxation times relative to the control, whereas in NPS gels, the presence of additives markedly reduced T_2_ values (Table 1). These contrasting effects suggest that the additives interfere with the molecular dynamics of water differently depending on the amylose content of the starch used for gel formation. The results point to a modified water–starch interaction profile, which may be critical for network stability and retrogradation behavior. Importantly, the use of mean correlation times appears to provide a more sensitive and informative descriptor of water mobility in starch-based systems during storage than traditional reliance on T_1_ and T_2_ relaxation times alone, offering valuable methodological implications for the characterization of hydrocolloid matrices.

Recently, research confirmed that buckwheat husk fiber (BH), which is rich in total fiber (TDF 71.40 g/100 g, including 61.06% insoluble fiber (IDF) and 10.34% soluble fiber (SDF)), is important for stabilizing starch matrices. BH acts by competing with amylose for water, which inhibits granule swelling and reduces amylose leakage. In native starch (PS/NPS), adding BH decreased the breakdown parameter (BD), an indicator of enhanced starch stability, and slowed retrogradation in PS gels, resulting in less increase in hardness. BH had a stronger effect on PS than on WPS, confirming its greater interaction with amylose. Rheologically, adding BH to PS and WPS pastes lowered the consistency coefficient (K). Additionally, in 5% PS pastes, BH reduced the hysteresis loop area by 50%, demonstrating greater rheological stability of PS/BH systems compared to WPS/BH. When compared with other additives, buckwheat husk fiber at 0.2% in mixtures with waxy potato starch (WPS) had less impact on maximum viscosity than apple fiber (AF) and oat fiber (OF), likely due to differences in soluble and insoluble fiber content. Oat fiber (OF) exhibited greater rheological stability, indicated by a smaller total hysteresis loop area, in WPS than apple fiber (AF) [61,62].

It is also important to consider the impact of other functional additives, such as apple juice (AJ), on water binding and starch gelatinization. Recent research has shown that adding apple juice (pH 3.45) to potato starch (PS) mixtures delays the gelatinization process (raising T_0_ from 61.2 °C to 70.1 °C) and greatly decreases the maximum viscosity (ηmax) of PS pastes (by nearly 75%). This effect is linked to acid hydrolysis in an acidic environment, which reduces viscosity by breaking down amorphous regions of starch granules, outweighing the influence of buckwheat fiber (BH) [61,62].

**Figure 2:**
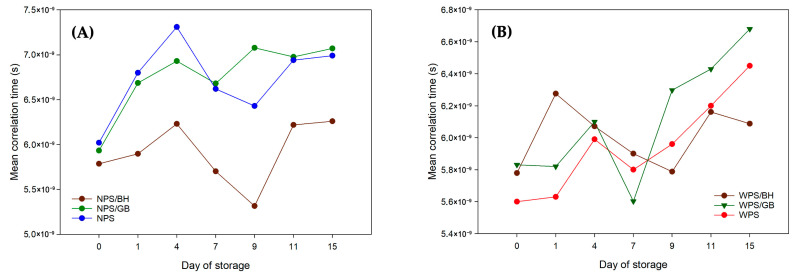
Mean correlation times of (**A**) NPS, NPS/BH, and NPS/GB; (**B**) WPS, WPS/BH, and WPS/GB.

Changes in mean correlation times in WPS gels supplemented with BH and GB demonstrated that the retrogradation process after seven days of storage is strongly dependent on the presence of the respective additive. Incorporation of BH resulted in lower mean correlation times compared to control gels (Figure 2), indicating weaker water-polymer interactions [63,64]. This effect was associated with increased molecular mobility of water molecules and reduced structural ordering within the gel matrix. In contrast, the addition of the barley-derived preparation (GB) led to an increase in mean correlation times, suggesting enhanced molecular ordering and a more stabilized gel structure. In NPS gels, the addition of GB exhibited only a minor effect on mean correlation times, suggesting that green barley does not exert a significant influence on the structural reorganization typically associated with the retrogradation process. In contrast, supplementation with BH markedly reduced mean correlation times, indicating that BH in combination with NPS hinders the molecular ordering of the system. The conducted analyses demonstrated that the addition of buckwheat hull preparation effectively inhibited conformational changes associated with water binding in waxy starch gels over a two-week storage period, compared to the changes observed in normal potato starch gels. An opposite effect was found when green barley was incorporated, where both starch systems exhibited distinct structural responses. In waxy starch gels, a greater restriction of water molecular mobility was observed compared with normal starch gels, which can be attributed to the higher proportion of branched amylopectin chains [64].

The literature shows that additives like green barley and buckwheat husks affect the structural and functional properties of starch gels, but their effects differ depending on the type of starch and additive used. For example, adding buckwheat husk to starch gels increases resistant starch, phenols, and antioxidant activity, while also reducing the product’s glycemic response. Additionally, BH supplementation effectively inhibits conformational changes related to water binding in starch gels during storage, which may help decrease retrogradation and improve gel stability [65]. It was also found that the addition of GB has only a slight effect on average correlation times, suggesting no significant impact on the structural reorganization associated with retrogradation. However, the inclusion of GB causes different structural responses in waxy and regular starch gels, which may be due to differences in the structures of amylopectin and amylose [66].

## 4. Conclusions and Future Perspectives

Amylose, a linear glucose polymer, and amylopectin, its highly branched counterpart, jointly determine the physicochemical properties of starch. Their ratio strongly affects water affinity, solubility, and molecular interactions within starch systems. LF NMR analysis confirmed that increasing amylopectin content, as in waxy starches, enhances water mobility due to the higher proportion of α-1,6-glycosidic linkages.

The study demonstrated that the addition of buckwheat husk markedly inhibited conformational changes related to water binding in waxy starch gels during two weeks of storage, in contrast to the effects observed in normal potato starch gels. Conversely, supplementation with green barley produced the opposite trend, with waxy starch gels exhibiting greater restriction of water molecular dynamics than their normal starch counterparts. This phenomenon is attributed to the highly branched structure of amylopectin.

The insoluble fiber fraction present in buckwheat husk appears to interact with amylopectin, entrapping water molecules within the polymeric network. In contrast, green barley, owing to its hydrophilic nature, preferentially binds water associated with amylose structures.

From a technological perspective, these findings provide valuable insight into the targeted modulation of starch–water interactions in food systems. Incorporation of plant-based additives such as buckwheat husk or green barley may be strategically applied to control gel structure, texture, and stability during storage. This knowledge can be harnessed in the development of innovative starch-based products with tailored functional properties, extended shelf life, and enhanced nutritional value.

## Figures and Tables

**Table 1:** Spin–lattice and spin–spin relaxation times for investigated samples.

Sample	Relaxation Time (ms)	Day of Storage					
		0	1	4	7	11	15
WPS	T_1_	1478 ± 2	1476 ± 1	1433 ± 2	1440 ± 2	1498 ± 1	1509 ± 2
	T_2_	900 ± 4	910 ± 3	990 ± 4	991 ± 4	961 ± 4	867 ± 4
WPS/BH	T_1_	1723 ± 3	1767 ± 2	1770 ± 1	1722 ± 1	1713 ± 2	1733 ± 2
	T_2_	1014 ± 5	977 ± 4	1004 ± 3	998 ± 3	1007 ± 5	972 ± 5
WPS/GB	T_1_	1806 ± 2	1807 ± 2	2029 ± 2	1969 ± 2	2169 ± 2	2130 ± 1
	T_2_	1056 ± 5	1058 ± 4	1058 ± 4	1185 ± 4	1196 ± 3	1127 ± 3
NPS	T_1_	1567 ± 3	1539 ± 1	1612 ± 1	1655 ± 1	1699 ± 1	1720 ± 2
	T_2_	1142 ± 6	1160 ± 3	1122 ± 3	1082 ± 5	1012 ± 4	999 ± 4
NPS/BH	T_1_	1738 ± 1	1709 ± 1	1847 ± 1	1750 ± 2	1783 ± 2	1772 ± 6
	T_2_	1022 ± 3	991 ± 4	1027 ± 4	1040 ± 5	1113 ± 4	989 ± 4
NPS/GB	T_1_	1703 ± 2	1722 ± 1	1939 ± 2	1949 ± 1	1942 ± 2	1950 ± 1
	T_2_	983 ± 5	905 ± 4	989 ± 5	1025 ± 5	973 ± 4	989 ± 5

WPS—waxy potato starch; WPS/BH—waxy potato starch with buckwheat hull fiber; WPS/GB—waxy potato starch with green barley; NPS—non-waxy potato starch; NPS/BH—non-waxy potato starch with buckwheat hull fiber; NPS/GB—non-waxy potato starch with green barley.

## Data Availability

All data supporting the findings of this study are contained within the article.

## Abbreviations

The following abbreviations are used in this manuscript:

BH	Buckwheat hull fiber
BPP	Bloembergen–Purcell–Pound equations
GB	Green burley
FID	Free induction decay
LF NMR	Low-Field Nuclear Magnetic Resonance
NPS	Non-waxy potato starch
NPS/BH	Non-waxy potato starch with buckwheat hull fiber
NPS/GB	Non-waxy potato starch with green barley
WPS	Waxy potato starch
WPS/BH	Waxy potato starch with buckwheat hull fiber
WPS/GB	Waxy potato starch with green barley
